# The Book World of Medicine and Science

**Published:** 1899-12-23

**Authors:** 


### The Book World of Medicine and Science.

A Manual of Surgery for Students and Practitioners. By William Rose, M.B., B.S.Lond., F.R.C.S., and Albert Carless, M.S.Lond., F.R.C.S. Second edition. (London: Baillière, Tindall, and Cox. 1899. Price 21s. net.)

We have carefully looked through this handsome volume, and have nothing but praise for its contents. The changes in this edition are not very numerous, but they are all improvements, and as the book now stands it may be highly recommended as an admirable exposition of the present position of surgery and as a manual highly suited for the use of the student. Perhaps, indeed, it is more suited to the student than to the practitioner, but, in any attempt to compress the facts of surgery within the compass of a single volume, that seems inevitable. The fact is the practitioner does not want a manual but a book of reference, and if this is to be at all complete it must be a large one, whereas the student is always being taught in the wards, and what he wants is not so much a complete treatise as a handbook which shall put in order the wider knowledge which he is picking up from many sources while working in the hospital and the lecture theatre. It is method, careful condensation, and proper wording that the student wants, so as to enable him to focus his knowledge, and all this he gets in plenty in the book before us. There is no doubt that a very large proportion of its pages is devoted to operative surgery. But then surgery nowadays is operative if it is anything, and thus the book does but reflect the present position of the subject. It seems a pity that some of the purely surgical work which now falls, too often as we think, into the hands of the gynæcologist should not have been included in such a volume as this. We can see no excuse for the exclusion of ovariotomy and hysterectomy, and for there being no mention of operation for the treatment of intra-peritoneal hæmorrhage from ruptured ectopic gestation in a manual of surgery. All such proceedings are purely surgical, and, moreover, they are the sort of surgery from which no surgeon can wholly escape, however much he may regard gynæcology as a specialty outside his province. No doubt, as is stated in the preface, these things have been left out for want of room. But it is a pity, for we can hardly admit, as the preface suggests, that such subjects do not "encroach on the domains of general surgery." With this exception, however, nothing could be better for the purposes of the student than the work before us. It is not too large; it is well illustrated, for although some of the pictures are hardly to be commended as works of art they generally serve well to demonstrate the points at issue; and the teaching, while thoroughly up to date, is sound and solid, and such as is likely to be acceptable to examiners.

Lord Lister and Surgery. By Robert Turner, M.A., M.D., F.R.C.S.Edin. (London: H. Glaisher. 1899. Small 8vo. 28 pp. 1s.)

As an article in a lay monthly magazine these 28 pages would have been valuable; they present writing which is concise, simple, and not too technical, and a general history of the advance of modern surgery which is correct, and, within its limits, judicial. But we ask, *cui bono?* The younger man does not need such a summary as this; the older man—if he is attempting to improve his surgical technique still—can learn nothing of new technique from it. The anecdote on page 14 affords an excellent text which the author might have pressed to a practical conclusion. If every country surgeon who has to take care of his own instruments made a rule of boiling once a week in a soda solution every instrument which he has used in the previous seven days, he would save an infinite number of suppurating wounds and an infinity of labour to himself.

London University Guide and University Correspondence College Calendar, 1899—1900. (London: University Correspondence College Press. Small 8vo. 64 pp.)

The University Correspondence College is now a well-established institution, doing excellent work in preparing candidates for the London University examinations. Naturally, this method of teaching is confined to examinations in arts and mathematics, but, by arrangement with the University Tutorial College, the necessary laboratory accommodation for such examinations as the Prel. Sci. and Inter-Sci., or even the B.Sc., is provided. The examination results of the Correspondence College maintain the high level they reached some years ago. This guide is full of valuable hints as to books suitable for the various London University examinations, and will altogether be found a most useful manual for any student entering on the curriculum of the University.

Baillière, Tindall, and Cox.

